# An Evaluation of Dual Systems Theories of Adolescent Delinquency in a Normative Longitudinal Cohort Study of Youth

**DOI:** 10.1007/s10964-021-01433-z

**Published:** 2021-04-15

**Authors:** Aja Louise Murray, Xinxin Zhu, Jessica Hafetz Mirman, Denis Ribeaud, Manuel Eisner

**Affiliations:** 1 Department of Psychology, University of Edinburgh, Edinburgh, UK; 2 Department of Clinical and Health Psychology, University of Edinburgh, Edinburgh, UK; 3 Jacobs Center for Productive Youth Development, University of Zurich, Zurich, Switzerland; 4 Institute of Criminology, University of Cambridge, Cambridge, UK

**Keywords:** Adolescent risk-taking, Dual systems theory, Delinquency, Sensation-seeking, Self-regulation

## Abstract

Dual systems theories of adolescent risk-taking propose that the socioemotional and self-regulation systems develop at different rates, resulting in a peak in sensation-seeking in adolescence at a time when self-regulation abilities are not yet fully mature. This “developmental imbalance” between bottom-up drives for reward and top-down control is proposed to create a period of vulnerability for high-risk behaviors such as delinquency, substance use, unprotected sex, and reckless driving. In this study, data from the Swiss longitudinal normative z-proso study (*n* = 1522, *n* = 784 male; aged 11, 13, 15, 17, and 20) were used to test whether the presence of a developmental imbalance between sensation-seeking and self-regulation is associated with trajectories of engagement in delinquency across early adolescence to adulthood. Using a latent class growth analysis of sensation-seeking, self-regulation, and delinquency, it was found that a model with 3 classes was optimal in the whole sample and male sub-sample, including one class characterized by a developmental imbalance and corresponding adolescent peak in delinquency. In females, there was no evidence for a class that could be described according to the trajectories hypothesized in dual systems theory. This study’s results support the claim that a developmental imbalance may drive an adolescent increase in delinquency. However, this applies only to a small subgroup of individuals, particularly males.

## Introduction

There are varying definitions of “risky” behavior; however, developmental psychological theories of adolescent risk-taking typically define “risky” behavior, as any behavior that carries some possibility of a negative outcome, including health or social consequences (Romer and Khurana 2020). Delinquency is considered a prototypical example of such behavior, involving potential costs (e.g., criminal justice involvement, sanctions by schools) and rewards (e.g., thrills and social approval) (Burt and Simons 2013). It also shows a peak in adolescence, making it a prime candidate behavior to be explained by models of adolescent risk-taking (Barbot and Hunter 2012). Dominant among adolescent risk-taking models are dual systems models which propose that a developmental imbalance between socioemotional system and cognitive control system traits underlies a generalized increase in risk-taking during adolescence (Steinberg et al. 2008). These models have undergone considerable empirical evaluation in relation to a range of risk-taking behaviors (Shulman et al. 2016). However, their direct application to delinquency as a specific manifestation of risk-taking has been limited (see e.g., Cauffman et al. 2016). In this study, longitudinal data spanning ages 11–20 years was thus used to examine the claims of dual systems theory in relation to delinquency.

### Dual Systems Theories of Adolescent Risk-taking

Dual systems theories (Steinberg et al. 2008) have become the dominant paradigm for understanding an apparent increase in risk-taking in adolescence. In these models, the socioemotional system underlying sensation-seeking are assumed to mature faster than the cognitive control system underlying self-regulation. In fact, reward processing in the socioemotional system is assumed to show an activity peak in adolescence, while the cognitive control system is still developing. The result of this developmental mis-match is that during adolescence, individuals are particularly vulnerable to engaging in risk-taking behaviors such as unprotected sex, reckless driving, substance misuse, and delinquency as bottom-up reward drives are inadequately countered by top-down cognitive control.

Dual systems theory has also been noted as a promising development of criminological theories focused on the role of self-control in delinquency (Vazsonyi and Ksinan 2017). One of the most historically dominant theories of offending has been the general theory of crime (Gottfredson and Hirschi 1990), which proposes that low self-control is an early-determined, relatively stable, and unitary construct and a primary driver of criminal activity. More recent investigations have suggested that, in fact, self-control can be divided into multiple sub-components, some of which may map to the constructs of dual systems theory and show considerable and dissociable mean-level change over development (e.g., Forrest et al. 2019).

### Operationalizing Dual Systems Theories

Several previous reviews have evaluated dual systems theories, noting that empirical data supports the hypothesized developmental trajectories of sensation-seeking (inverted U-shaped) and self-regulation (monotonically increasing) and this pattern can be observed across different cultures (Steinberg et al. 2018). However, they have also noted some problems, such as the fact that many risk-taking behaviors peak after adolescence, perhaps due to opportunity factors (Defoe et al. 2019). A recurring concern has been the lack of specificity of dual system theory predictions and associated weak operationalization in empirical tests (Pfeifer and Allen 2016). For example, many studies have relied on cross-sectional data despite the fact that developmental imbalances are inherently longitudinal. Further, the operationalization of an *imbalance* between socioemotional and self-regulation systems has been criticized as many previous have either neglected to operationalize this important construct and/or used suboptimal methods to so do (Meisel et al. 2019). These suboptimal methods have included the use of observed difference scores, regression residuals, and moderated multiple regression. The first two methods are based on deriving differences between socioemotional and self-regulation system scores (either by subtraction or using regression residuals from the regression of one score on the other) and predicting risk-taking from these, while the latter is based on testing an interaction between socioemotional and self-regulation scores in predicting some risk-taking outcome. These methods suffer from a range of difficulties including interpretational ambiguity, low reliability, and, typically also a lack of a developmental dimension due to estimation only as cross-sectional (as opposed to longitudinal) constructs. As such, none of the methods that have been commonly used in previous studies have provided an unambiguous test of the idea that a developmental imbalance drives risk-taking. With the goal of improving the strength of empirical tests of dual systems theories, and with a particular focus on the challenge of operationalizing a developmental imbalance between self-regulation and socioemotional systems, a recent study offered two alternative recommendations to the above-mentioned suboptimal methods: longitudinal latent difference score modeling and growth mixture modeling (Meisel et al. 2019).

These recommendations are based on the idea that given that there are individual differences in developmental trajectories of sensation-seeking, self-regulation, and their extent of developmental imbalance (Mills et al. 2014), dual systems models suggest that adolescents with the largest developmental imbalance should show the greatest risk-taking concurrent with this imbalance. The proposed latent difference score modeling approach was based on examining the correlation between the developmental trajectories of the magnitude of imbalance between sensation-seeking and self-regulation and of risk-taking. The growth mixture modeling approach was based on identifying subgroups of individuals with different patterns/magnitudes of developmental imbalance and examining whether they mapped to greater risk-taking around periods of imbalance. An advantage of this latter approach is that it can parse the heterogeneity in risk-taking constructs into possible sub-groups. This feature is important because recent developments in adolescent risk-taking models have proposed that only a sub-group of adolescents with pre-existing self-regulation deficits may show a developmental imbalance and attendant maladaptive risk-taking (Romer et al. 2017). This is also consistent with developmental criminology frameworks which propose sub-groups of individuals following distinguishable delinquency trajectories (e.g., Moffitt 2017). In dual taxonomy theory, for example, one high risk “life-course persistent” sub-group is proposed to show the most persistent and problematic offending behavior, while others show a transient and relatively harmless increase during adolescence.

Both in general risk-taking and in delinquency in particular, only a handful of evaluations have used these newly recommended approaches. Most previous evaluations have relied on moderated multiple regression or observed difference score modeling (e.g., Rhodes et al. 2013), or examined sensation-seeking and self-regulation relations to delinquency but not their imbalance (e.g., Armstrong et al. 2020). Only two previous studies applied these recommended models, focusing specifically on substance use. One study found no evidence of a link between extent of developmental imbalance and adolescent substance use (Meisel et al. 2019). The other found no evidence for the joint self-regulation and sensation-seeking trajectories predicted by two major developmental imbalance models, namely, dual systems theory and the maturational imbalance model; however, they did find that members of the subgroup with the largest developmental imbalance between these two traits showed the highest levels of adolescent substance use (Meeus et al. 2021). No study has applied these recommended approaches to examining a dual systems model of delinquency.

Previous studies have sometimes implicitly considered “risk-taking” as though it represents a unitary construct explainable by a single theory of adolescent risk-taking; however, there is growing evidence that different forms of risk-taking and their sequelae show different developmental patterns, individual risk factors, and environmental constraints. For example, while delinquency peaks in adolescence, binge-drinking, fatalities due to drink-driving, substance use, sexually transmitted infections, and unintentional injuries, all tend to peak around or shortly after the transition to adulthood (see e.g., Willoughby et al. 2013). It has been speculated that delinquency may show this earlier peak because it is less constrained by a need for access to adult status, roles, and resources as compared to behaviors such as driving or substance use which are subject to age restrictions (Duell et al. 2018). It has also been noted that delinquency imposes a particularly significant cost on others (crime victims) compared to other risk-taking behaviors and thus may be more closely linked to callous-unemotional traits than other forms of risk-taking (Armstrong et al. 2020). These considerations suggest that delinquency as a specific form of risk-taking merits individual attention and it cannot be assumed that findings on other forms or risk-taking necessarily generalize to its explanation in a straightforward manner.

It is also important to consider the role of sex and gender in dual systems theories. Male adolescents are known to show considerably greater engagement in risk-taking in general (Duell et al. 2018), and offending in particular (Chen et al. 2015). Surprisingly, however, sex and gender differences have received little attention within a dual systems perspective. Exploration differences in the dual system model across males and females may, therefore, be a fruitful avenue for exploration to illuminate the significant over-representation of males in delinquency engagement in adolescence.

## Current Study

While dual systems theories are promising for illuminating engagement in delinquency in adolescence, their operationalization has been limited in previous research. Recent efforts to improve their operationalization have provided a means to accelerate progress in this area; however, thus far applications have been limited to two previous studies and none in delinquency. In this study, a dual systems theory model of adolescent delinquency was thus evaluated using a slight adaption of the recently recommended growth mixture model (Meisel et al. 2019) in a large longitudinal sample covering the entire period of adolescence (with data at ages 11, 13, 15, 17, and 20). Following from the dual systems theory claim that greater imbalances should lead to greater risk-taking, it was hypothesized that in *subgroups* where there was a greater developmental imbalance between sensation-seeking and self-regulation, this would be accompanied by higher concurrent levels of delinquency. Both whole-sample analyses and gender-stratified analyses were conducted; the latter to explore the possibility that there are gender differences in subgroups of joint dual system construct and delinquency developmental trajectories.

## Methods

### Participants

Data came from the age 11, 13, 15, 17 and 20 waves of the Zurich project on social development from childhood to adulthood (z-proso, http://www.cru.ethz.ch/en/projects/z-proso.html), a longitudinal study of psychosocial development with a particular focus on crime and delinquency (Eisner et al. 2018). The z-proso sample was from 56 primary schools (Zurich, Switzerland), selected using a stratified random sampling approach based on school size and location. According to the developmental period of interest, the current study focuses on the latest five assessment waves (i.e., wave 4–8), including 1522 young people (approximately 50% male, see Table 1, for demographic information), from the initial target sample *n* = 1675 (for details on the non-response and attrition, see Eisner et al. 2019). Recruitment and assessment procedures, and demographic characteristics for the z-proso have been discussed in detail previously (Eisner et al. 2019).

**Table 1:** Sample demographics (*n* = 1522)

	Frequency	Percentage
*Gender*		
Male	784	51.5%
Female	738	48.5%
^a^ *Nationalities*		
Switzerland	745	48.9%
Serbia-Montenegro	103	6.8%
Portugal	88	5.8%
Sri Lanka	82	5.4%
Germany	57	3.7%
Italy	54	3.5%
Turkey	52	3.4%
^b^*Parental SES*		
	44.55 (*Mean*)	17.74 (*SD*)

^a^The sample is ethnically diverse with 70 nationalities represented in the baseline sample of the z-proso study; only the most common are shown

^b^Parental SES is based on the international socio-economic index of occupational status (ISEI)

### Ethics

Ethical approval for this study was granted by the Ethics Committee from the Faculty of Arts and Social Sciences of the University of Zurich. For each round of data collection, informed consent was collected as appropriate (i.e., from participants at age 13 onwards or their parents when they were younger).

### Measures

#### Dual systems components

Sensation-seeking and self-regulation were measured using Grasmick’s self-control scale, which is based on Gottfredson and Hirschi’s (1990) low self-control theory of crime. The measure acknowledges the multi-dimensional nature of self-control (Wojciechowski 2020), measuring six subdimensions in total. Two of these subdimensions have been noted to capture constructs that map to the constructs of dual systems theory (e.g., Forrest et al. 2019). Specifically, sensation-seeking was measured using the 2-item risk-seeking subscale from an adapted version of Grasmick’s self-control scale (Grasmick et al. 1993). The two items were: “Excitement and adventure are more important to me than security” and *“*Sometimes I do dangerous things just for the fun of it”. Item responses were recorded on a 4-point scale from 1 = *false* to 4 = *true* and summed to provide an overall sensation-seeking score with a possible range from 2–8. Self-regulation was measured using the 2-item impulse control subscale from the same adapted version of Grasmick’s self-control scale (Grasmick et al. 1993). The two items were “I often act on the spur of the moment without stopping to think” and “I often do whatever brings me pleasure here and now, even at the cost of some distant goal”. Item responses were recorded on a 4-point scale from 1 = *false* to 4 = *true*. Scores were reversed and summed to provide an overall self-regulation score, with a possible range from 2–8. Previous factors analyses in the current and other samples have supported the proposed multi-dimensional structure of Grasmick’s self-control scale and support making a distinction between the sensation-seeking and self-regulation components (Ribeaud and Eisner 2006).

#### Delinquency

Delinquency was measured using a variety score comprising responses to items describing 7 delinquent behaviors that were measured consistently across all waves included in the present study. Variety scores are considered advantageous over alternative methods of scoring delinquency (e.g., frequency-based scores) because they avoid the problem of scores being largely driven by frequent minor offences (Sweeten et al. 2013). These items referred to stealing at home, shoplifting goods worth less than 50CHF, shoplifting goods worth more than 50CHF, vehicle theft, fare dodging, vandalism, and assault. The 12-month incidence for each item was measured on a binary response format and a sum of these responses was used to create a composite score for each wave. As such, greater values on the score indicate engagement in a greater variety of delinquent behaviors, with a possible score range from 0 to 7 delinquent behaviors.

### Statistical Methods

To test a dual systems theory account of adolescent delinquency, a longitudinal latent class growth analysis approach was used (Meisel et al. 2019). In this approach, the joint longitudinal trajectories of sensation-seeking, self-regulation, and delinquency were estimated. Latent class models for between 1 and 8 classes were fitted, with an upper limit of 8 used to preserve parsimony. These models were firstly fitted for the whole sample and in separate gender-stratified subsamples. As compared to alternative approaches of examining gender differences (e.g., predicting class membership from gender) this was considered optimal because it allows for the possibility that different models would be optimal for males or females (Murray et al. 2019). Gender-stratified analyses were used rather than a multi-group model because this allowed for the possibility that a different number of classes would be optimal to characterize the joint trajectories of males versus females.

A Lo-Mendall-Rubin (LMR) test was used to determine an optimal number of classes, with information theoretic criteria used as supplementary information to guide model selection where the LMR was not definitive. Linear and quadratic growth factors were specified in these models, reflecting previous findings that all three constructs show curvilinear growth over development. Intercept factor loadings were fixed to 1, linear slope factor loadings were fixed proportional to the distance between waves based on the median ages at each measurement occasion, and quadratic factor loadings were fixed equal to the square of the slope factor loadings. Growth factor variances (and by implication factor covariances) were fixed to zero within each class. This reflects an assumption that the classes do not capture “true” classes, but are the result of convenient discretization of a continuous distribution. Models were fit in Mplus 8.4 (Muthén and Muthén 2015), with robust maximum likelihood (MLR) estimation. The full information maximum likelihood (FIML) approach was applied to handle missing data. This provides unbiased parameter estimated provided data are missing at random (MAR) in Rubin’s (1976) terms (i.e., that the probability of missingness is independent of the missing values conditional on the variables included in the model). The growth trajectories within the resultant classes from the optimal model were inspected to determine whether classes with a greater developmental imbalance between sensation-seeking and self-regulation around adolescence show a greater peak in delinquency around this time.

## Results

### Descriptive Statistics

**Fig. 1:**
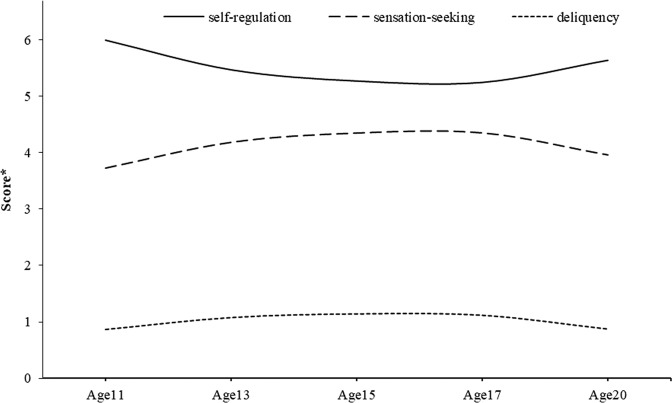
Average model-based trajectories of self-regulation, sensation-seeking, and delinquency. A higher score* indicates a higher level of self-regulation/sensation-seeking or engaging a greater variety of delinquency

The descriptive statistics for the study variables are provided in Table 2. These suggest a peak in sensation-seeking and delinquency at age 15. Self-regulation also showed a dip in mid-adolescence. The average model-based trajectories of these constructs from a parallel process latent growth curve model are shown in Fig. 1.

**Table 2:** Descriptive statistics

Construct	*N*	*Mean*	*SD*	*min*	*max*
Age 11 sensation-seeking	1134	3.69	1.55	2	8
Age 13 sensation-seeking	1346	4.24	1.54	2	8
Age 15 sensation-seeking	1426	4.40	1.46	2	8
Age 17 sensation-seeking	1279	4.25	1.47	2	8
Age 20 sensation-seeking	1179	4.00	1.41	2	8
Age 11 self-regulation	1128	6.05	1.34	2	8
Age 13 self-regulation	1340	5.41	1.22	2	8
Age 15 self-regulation	1437	5.26	1.13	2	8
Age 17 self-regulation	1289	5.34	1.20	2	8
Age 20 self-regulation	1179	5.61	1.22	2	8
Age 11 delinquency	1132	0.83	1.01	0	6
Age 13 delinquency	1350	1.15	1.11	0	7
Age 15 delinquency	1442	1.19	1.07	0	7
Age 17 delinquency	1299	1.02	1.05	0	6
Age 20 delinquency	1175	0.90	0.95	0	6

### Latent Class Growth Analysis

**Fig. 2:**
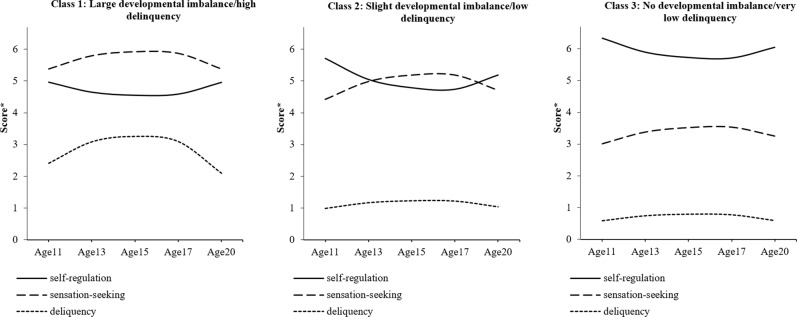
3-class model (whole sample). A higher score *indicates a higher level of self-regulation/sensation-seeking or engaging a greater variety of delinquency

The model fits, LMR test statistics, and entropy values for the 1–8 class models are provided in Table 3. The LMR test suggested that a 3-class model was optimal. Model parameters for the 3-class model are provided in Table 4 and visualized in Fig. 2. The models are plotted on an unstandardized scale and can thus be interpreted in the units of the original scores (where sensation-seeking and self-regulation have a possible range from 2–8 and delinquency has a possible range from 0–7 types of delinquent act). Full output for all models is provided at: https://osf.io/mb4w2/. The first class (7.4% of the sample) showed a mid-adolescent peak in both sensation-seeking and delinquency and relatively stable levels of self-regulation. This class had the highest levels of delinquency (peaking at ~3 types of delinquent acts) and sensation-seeking and the lowest levels of self-regulation overall. This class was, therefore, labeled “large developmental imbalance/high delinquency”. The second class (38.7%) had stably low levels of delinquency (stable at ~1 type of delinquent act) with only a very subtle elevation of offending behavior around mid-adolescence accompanied by a much more pronounced peak in sensation-seeking and dip in self-regulation. The imbalance between these latter two traits was, however, only small compared to the first group. This class was labeled “slight developmental imbalance/low delinquency”. The third class showed consistently very low levels of delinquency (consistently <1 type of delinquent act), consistently low levels of sensation-seeking (showing a slight peak in mid-adolescence), and consistently high levels of self-regulation. This class was labeled “no developmental imbalance/very low delinquency”.

**Table 3:** Model fits for the 1–8 class models for the whole sample

Model	LMR	*p*	AIC	BIC	saBIC	Entropy
1-class	–	–	62421.903	62549.770	62473.528	N/A
2-class	2756.660	<0.001	59647.624	59828.769	59720.759	0.818
**3-class**	**2756.660**	**<0.001**	**58807.401**	**59041.823**	**58902.046**	**0.821**
4-class	388.965	0.114	58433.127	58720.827	58549.283	0.762
5-class	305.980	0.069	58142.971	58483.949	58280.638	0.785
6-class	209.934	0.567	57950.172	58344.428	58109.349	0.795
7-class	157.194	0.558	57810.834	58258.367	57991.521	0.784
8-class	157.243	0.156	57671.445	58172.257	57873.643	0.789

Solution(s) considered “best-fitting” indicated in bold

**Table 4:** Model parameters for optimal (3-class) longitudinal latent growth analysis model (whole sample: *n* = 1522)

Classes			Self-regulation			Sensation-seeking			Delinquency		
Class	Class label	Size	Intercept (SE)	Linear slope (SE)	Quadratic slope (SE)	Intercept (SE)	Linear slope (SE)	Quadratic slope (SE)	Intercept (SE)	Linear slope (SE)	Quadratic slope (SE)
1	Large developmental imbalance/high delinquency	7.4%	4.959 (0.171)	−1.667 (0.645)	1.662 (0.546)	5.381 (0.193)	2.204 (0.692)	−2.204 (0.611)	2.411 (0.256)	3.675 (1.040)	−3.988 (0.949)
2	Slight developmental imbalance/low delinquency	38.7%	5.710 (0.082)	−3.288 (0.272)	2.769 (0.226)	4.426 (0.127)	2.848 (0.318)	−2.565 (0.269)	0.982 (0.069)	0.970 (0.220)	−0.917 (0.188)
3	No developmental imbalance/ very low delinquency	53.7%	6.340 (0.054)	−2.224 (0.226)	1.937 (0.192)	3.011 (0.059)	1.861 (0.224)	−1.619 (0.198)	0.583 (0.032)	0.853 (0.129)	−0.847 (0.117)

**Fig. 3:**
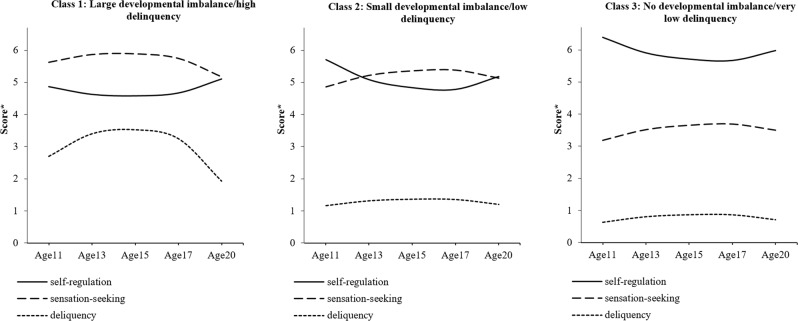
3-class model (males). A higher score* indicates a higher level of self-regulation/sensation-seeking or engaging a greater variety of delinquency

**Fig. 4:**
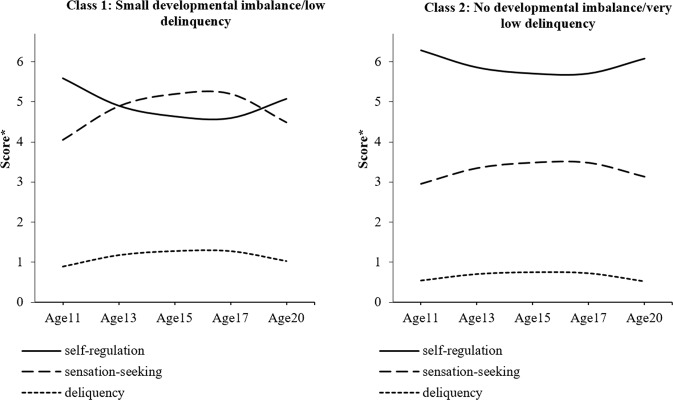
2-class model (females). A higher score* indicates a higher level of self-regulation/sensation-seeking or engaging a greater variety of delinquency

**Fig. 5:**
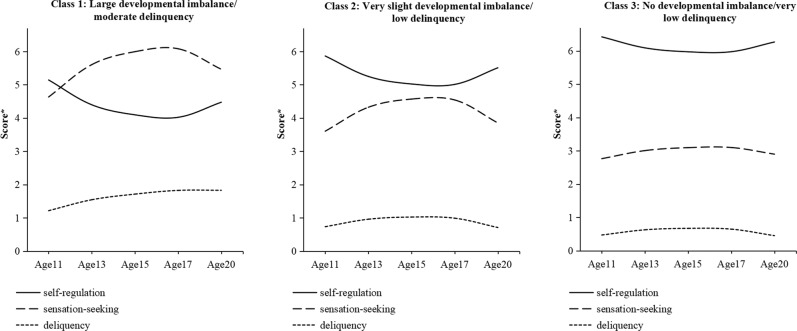
3-class model (females). A higher score* indicates a higher level of self-regulation/sensation-seeking or engaging a greater variety of delinquency

The statistical fit indices for gender-stratified analyses are shown in Table 5. For males, a 3-class model was determined as the optimal model according to the LMR test. For females, the LMR test indicated that a 2-class model fit the data best. However, the information statistics AIC, BIC, and saABIC also provided support for a 3-class model because they declined sharply from a 2-class to 3-class model. Moreover, the 3-class (compared to 2-class) model included a meaningful subgroup with a high level of delinquency, and its trajectory differed from that of the whole sample. Therefore, both 2- and 3-class models were retained. The models are summarized Tables 6 and 7 and Figs. 3, 4, and 5.

**Table 5:** Model fits for the 1–8 class models for the male and female samples

Model	LMR	*p*	AIC	BIC	saBIC	Entropy
*Males*						
1-class	–	–	32988.647	33100.592	33024.380	–
2-class	1400.401	<0.001	31587.232	31745.822	31637.855	0.806
**3-class**	**402.361**	**0.032**	**31198.834**	**31404.068**	**31264.345**	**0.817**
4-class	176.766	0.293	31039.415	31291.293	31119.816	0.829
5-class	140.882	0.416	30916.419	31214.941	31011.709	0.751
6-class	120.209	0.419	30814.407	31159.573	30924.586	0.770
7-class	83.253	0.345	30749.905	31141.715	30874.973	0.761
8-class	65.645	0.319	30703.275	31141.729	30843.232	0.745
*Females*						
1-class	–	–	28477.103	28587.598	28511.389	–
**2-class**	**1155.331**	**0.001**	**27324.277**	**27480.811**	**27372.850**	**0.765**
**3-class**	**396.408**	**0.090**	**26941.867**	**27144.440**	**27004.725**	**0.792**
4-class	179.905	0.198	26779.237	27027.850	26856.382	0.797
5-class	119.473	0.772	26677.955	26972.608	26769.386	0.791
6-class	103.583	0.410	26592.804	26933.496	26698.520	0.784
7-class	89.191	0.261	26522.263	26908.994	26642.265	0.770
8-class^a^	62.633	0.526	26478.681	26911.452	26612.969	0.761

Solution(s) considered “best-fitting” indicated in bold

^a^Estimation difficulties were encountered in this model

**Table 6:** Model parameters for optimal (3-class) longitudinal latent growth analysis model (males: *n* = 784)

Classes			Self-regulation			Sensation-seeking			Delinquency		
Class	Class label	Size	Intercept (SE)	Linear slope (SE)	Quadratic slope (SE)	Intercept (SE)	Linear slope (SE)	Quadratic slope (SE)	Intercept (SE)	Linear slope (SE)	Quadratic slope (SE)
1	Large developmental imbalance/ high delinquency	9.9%	4.867 (0.222)	−1.336 (0.868)	1.573 (0.736)	5.627 (0.310)	1.416 (0.845)	−1.864 (0.729)	2.693 (0.378)	3.975 (1.434)	−4.746 (1.174)
2	Small developmental imbalance/low delinquency	40.3%	5.703 (0.120)	−3.078 (0.407)	2.559 (0.325)	4.857 (0.149)	1.794 (0.484)	−1.522 (0.391)	1.170 (0.114)	0.782 (0.398)	−0.744 (0.188)
3	No developmental imbalance/very low delinquency	49.9%	6.389 (0.083)	−2.410 (0.328)	1.999 (0.277)	3.181 (0.089)	1.652 (0.327)	−1.334 (0.282)	0.634 (0.050)	0.875 (0.222)	−0.796 (0.200)

**Table 7:** Model parameters for optimal (2-and 3-class) longitudinal latent growth analysis model (females: *n* = 738)

Classes			Self-regulation			Sensation-seeking			Delinquency		
Class	Class Label	Size	Intercept (SE)	Linear slope (SE)	Quadratic slope (SE)	Intercept (SE)	Linear slope (SE)	Quadratic slope (SE)	Intercept (SE)	Linear slope (SE)	Quadratic slope (SE)
*2-class model*											
1	Small developmental imbalance/low delinquency	37.2%	5.595 (0.122)	−3.437 (0.374)	2.924 (0.317)	4.045 (0.166)	4.298 (0.412)	−3.859 (0.343)	0.894 (0.072)	1.466 (0.276)	−1.329 (0.242)
2	No developmental imbalance/very low delinquency	62.8%	6.281 (0.077)	−2.162 (0.302)	1.957 (0.256)	2.954 (0.086)	1.986 (0.332)	−1.810 (0.295)	0.544 (0.044)	0.857 (0.148)	−0.878 (0.133)
*3-class model*											
1	Large developmental imbalance/moderate delinquency	9.5%	5.143 (0.218)	−3.703 (0.764)	3.034 (0.665)	4.633 (0.283)	4.843 (0.756)	−4.011 (0.645)	1.216 (0.232)	1.524 (0.687)	−0.911 (0.660)
2	Very slight developmental imbalance/low delinquency	50.4%	5.864 (0.099)	−3.122 (0.304)	2.767 (0.260)	3.605 (0.127)	3.707 (0.383)	−3.458 (0.317)	0.735 (0.052)	1.191 (0.209)	−1.216 (0.183)
3	No developmental imbalance/very low delinquency	40.0%	6.425 (0.090)	−1.692 (0.373)	1.538 (0.327)	2.766 (0.086)	1.233 (0.337)	−1.098 (0.303)	0.472 (0.058)	0.829 (0.168)	−0.849 (0.148)

In the male subsample, the classes were very similar to those that emerged in the full sample analyses. The first class was characterized by a developmental imbalance in sensation-seeking and self-regulation accompanied by a mid-adolescent peak in delinquency (peaking at ~3 types of delinquent act). This class was thus labeled “large developmental imbalance/high delinquency”. This class accounted for 9.9% of the sample. The second class (40.3% of the sample) was characterized by an adolescent sensation-seeking peak and a self-regulation dip; however, this created only a small developmental imbalance compared to the first group and it was not accompanied by a substantive escalation of delinquency (stably ~1 type of delinquent act). It was, therefore, labeled “small developmental imbalance/low delinquency”. The final class (49.9% of the sample) showed stably low levels of sensation-seeking, stably high levels of self-regulation and stably (very) low levels of delinquency (consistently <1 type of delinquent act over adolescent development). This class was, therefore, labeled “no developmental imbalance/very low delinquency”.

Given that the fit statistics indicated that either a 2- or 3-class model could represent an optimal model in females, both are interpreted. In the 2-class model, the first class (37.2% of the sample) was characterized by a mid-adolescent peak in sensation-seeking and dip in self-regulation. However, this was not accompanied by an escalation in delinquency (it remained consistently around 1 type of delinquent act over adolescence). This group was, therefore, labeled “small developmental imbalance/low delinquency”. The second group (62.8% of the sample), was characterized stably low levels of sensation-seeking, stably high levels of self-regulation, and very low levels of delinquency (<1 type of delinquent act) across adolescence. This class was, therefore, labeled “no developmental imbalance/very low delinquency”.

In the 3-class model in the female sub-sample, the first class (9.5% of the sample) was characterized by a relatively large sensation-seeking/self-regulation developmental imbalance but only a moderate increase in delinquency. In contrast to the male group showing a delinquency escalation, this was characterized by a steady curvilinear increase from age 11 rather than a pronounced peak around mid-adolescence. For this group, delinquency scores were initially ~1 type of delinquent act and had increased to ~2 types of delinquent act by age 20. The peak in sensation-seeking also occurred later in females as compared to the corresponding male group This group was thus labeled “large developmental imbalance/moderate delinquency”. The second class (50.4% of the sample) showed a mid-adolescent peak in sensation-seeking and corresponding dip in self-regulation. There was a very slight increase in delinquency corresponding to the point of maximal developmental imbalance but overall delinquency levels in this group were low (~1 type of delinquent act). Further, the developmental imbalance in this group was much smaller than in other groups showing an imbalance. This group was thus labeled “very slight developmental imbalance/low delinquency”. Finally, the third class (40.0% of the sample) had stably low levels of sensation-seeking, stably high levels of self-regulation, and only very low levels of delinquency (<1 type of delinquent act) at all stages of adolescence. This class was, therefore, labeled “no developmental imbalance/very low delinquency”.

## Discussion

Dual systems theory predicts that an escalation in delinquency in adolescence is driven by a developmental imbalance between self-regulation and sensation-seeking. The former is proposed to strengthen gradually over development and to not fully mature until adulthood whereas the latter is proposed to show a peak in reactivity in adolescence. This is proposed to result in a period in adolescence where the drive for novel/exciting experiences is insufficiently tempered by self-regulatory capacities. Previous studies have noted heterogeneity in the developmental trajectories of sensation-seeking, self-regulation, and their imbalance, as well as in the developmental trajectories of delinquency. However, the dual systems theory prediction that youth showing greater developmental imbalances also show higher levels of adolescent delinquency had yet to be tested. In this study, the goal was thus to provide an evaluation of the claim that a developmental imbalance between sensation-seeking and self-regulation traits is associated with a peak in offending behavior in adolescence, using an appropriate *developmental* operationalization of imbalance.

Using longitudinal latent growth analysis, the current study’s results suggested that a developmental imbalance accompanied by a delinquency peak accounted for only a small sub-group of youth (7.4%) in the sample. This group, labeled “large developmental imbalance/high delinquency”, showed the highest delinquency of all subgroups. Most youth showed no evidence of a pronounced adolescent peak in delinquency, even when a peak in sensation-seeking was evident (i.e., youth in the “slight developmental imbalance/low delinquency group”). Thus, these results indicate that the claims postulated by the dual systems model of risk-taking held only for a minority of youth, who exhibited the highest levels of offending; whereas the claims did not hold for the majority of youth who exhibited lower levels of offending. Gender-stratified analyses suggested, furthermore, that the model applies primarily to male youth (9.9% of males who fell into the “large developmental imbalance/high delinquency” group in the male subsample analyses). The implications of these findings include redefining the scope of the dual systems model of adolescent risk-taking from a theory of normative development towards a theory of developmental psychopathology.

This study’s results contribute important evidence to the debate regarding the universality of a dual systems model account of adolescent risk-taking. A number of previous reviews have noted that not all adolescents show a developmental imbalance in socioemotional and cognitive control systems nor an adolescent peak in risk-taking (Crone et al. 2016). The current study parsed this heterogeneity into possible sub-groups (also see Meisel et al. 2019). Doing so, it was observed that those youth with a larger developmental imbalance are the same who show a pronounced peak in a prototypical risk-taking behavior, namely, delinquency. The normative nature of the current sample (see Eisner et al. 2018) also allows an estimate of the prevalence of this high-risk sub-group, suggesting that this accounts for only a minority of youth (~7% of youth or ~10% of male youth). Taken together, these findings suggest that a dual systems model may provide an accurate account of adolescent risk-taking; however, only for a small at-risk sub-group.

The finding that a developmental imbalance and associated delinquency spike characterizes only a small sub-group of youth is in line with the Lifespan Wisdom Model (LWM). LWM builds on dual systems models but proposes that it describes only youth with pre-existing and stable deficits in cognitive control (Romer et al. 2017). In LWM, all youth are proposed to show a peak in sensation-seeking to facilitate exploration as part of healthy development; however, this creates a developmental imbalance and attendant maladaptive risk-taking for a minority of youth from whom the resultant exploratory drive is insufficiently supported by self-regulation abilities. While there is previous support for LWM, the majority of this evidence appears to have informed its development rather than having representing tests the predictions generated from the model in new data (Khurana et al. 2018). The current study, therefore, adds an important independent test of its predictions in new data. However, LWM does not make precise predictions about a number of core issues relating to developmental trajectories of delinquency, such as the size of the “at-risk” subgroup or possible distinctions between individuals who fall under the “low risk” majority. The current study’s findings provide some illumination on these issues that could be used to hone and increase the specificity of models such as LWM for testing in further independent research.

The current findings are also consistent with criminological evidence which suggests that there is a vulnerable sub-group of youth responsible for the majority of offending, engaging in more serious types of delinquency, and accounting for a disproportionate percentage of the societal costs of offending (Allard et al. 2014). A vulnerable sub-group of prolific offenders is also posited in dominant contemporary developmental criminology perspectives on delinquency. In dual taxonomy theory, for example, a “life-course persistent” group is proposed to show a stable and early emerging vulnerability for antisocial behavior due to early life transactions between neuropsychological difficulties and social-environmental factors such as harsh parenting (Moffitt 1993). This “at-risk” group is proposed to show self-regulation deficits from early in life; however, the role of an interaction of these deficits with a possible peak in sensation-seeking in adolescence has been little-discussed. The results from this study suggest that incorporating an interaction of this kind would be a fruitful extension of developmental criminology models in illuminating the causes of adolescent offending behavior in the “at-risk” group. The finding from the present study that self-regulation and sensation-seeking scores showed divergent developmental trajectories also contributes further support to criminological theories that suggest that self-control is not a unitary construct but includes multiple distinguishable dimensions (Ribeaud and Eisner 2006).

Exploratory gender-stratified analyses provided insights into the role of gender in joint developmental trajectories of sensation-seeking, self-regulation, and delinquency. These suggested that while males showed a sub-group characterized by a developmental imbalance and corresponding mid-adolescent peak in delinquency, females tended not to show any pattern consistent with a dual systems model (i.e., there was no group showing an adolescent peak in sensation-seeking and delinquency and a slight gradual growth in self-regulation). In the 3-class model, females showed one group (labeled “large developmental imbalance/high delinquency”) with a developmental imbalance between sensation-seeking and self-regulation but this occurred later than mid-adolescence and was not accompanied by a substantive peak in delinquency. The levels of delinquency for this sub-group were also overall lower than the corresponding male group showing a developmental imbalance. The other two groups for both males and females showed only minimal levels of delinquency and very little change in levels over adolescence and into adulthood and were, therefore, also not consistent with a dual systems theory model. Taken together, these analyses suggest that dual systems theories of adolescent risk-taking are primarily applicable to adolescent risk-taking in males.

There have been very few previous studies examining sex and gender differences within a dual systems framework. Some studies have identified sex or gender differences in sensation-seeking and self-regulation, with males tending to show higher levels of the former and lower levels of the latter but no substantive differences in their trajectories over time (Shulman et al. 2015). It has also been suggested that the influence of pubertal hormones on adolescent risk-taking may differ by sex. One study (Icenogle et al. 2017), for example, proposed that testosterone affects males’ socioemotional system such that it undermines their self-regulation system’s ability to inhibit reward impulses, whereas females may experience less affective arousal in relation to rewards and thus require less countervailing self-regulation. Finally, one study suggested a lack of gender differences on the Balloon Analogue Risk Task (BART) measure of risk-taking propensity and therefore concluded that gender differences in real-world risk-taking may be more related to differences in opportunity than underlying risk-taking propensities (Duell et al. 2018). In fact, the current study’s findings do not align precisely with these previous studies. They suggest that a subset of both males and females show a developmental imbalance consistent with that described by dual systems theory. However, only for males is this accompanied by an escalation in delinquency. It is possible that a developmental imbalance is expressed in different ways for females (e.g., via the emotional difficulties that are more common in adolescent females) and future studies examining a range of markers of adolescent functioning may provide further insights into possible differential expressions by gender.

The present study’s results also have implications for prevention, suggesting that developmental prevention programs for offending (see Farrington et al. 2017 for a review) would be best targeted specifically at youth who show low levels of self-regulation in combination with high sensation-seeking. This group already showed a profile of self-regulation and sensation-seeking that differed from that of the two low delinquency group at age 11 before the peak in delinquency occurred, suggesting that identification for intervention prior to an escalation of behavior problems is possible. For this group, prevention could address mitigating the impacts of a self-regulation vs sensation-seeking balance, such as through self-control training (Piquero et al. 2016) and/or channeling sensation-seeking into more “positive” forms of risk-taking (Duell and Steinberg 2019); however, future research will be beneficial to determine optimal intervention approaches for this group.

A further key area of future research concerns the generalizability of the current findings to other forms of risk-taking. Adolescent risk-taking models assume that delinquency shares a common basis with other forms of risk-taking such as substance misuse, reckless driving, sexual risk-taking, and self-harming behaviors (Romer et al. 2017). Thus, future research could assess whether similar subgroups emerge with respect to these outcomes. Similarly, delinquency is an umbrella term for a diverse set of behaviors and it will be important to determine whether some behaviors are particularly well or poorly described by the model in the current study, i.e., involving subgroups with and without a developmental imbalance between sensation-seeking and self-regulation. For example, distinctions are often made between aggressive and non-aggressive conduct problems and between reactive and instrumental aggression (Fairchild and Smaragdi 2018). It is possible that more impulsive and emotionally-hot forms of delinquency (e.g., reactive aggression) are better accounted for by the models of the current study (see e.g., Lickley and Sebastian 2018). Finally, our study focused on dual system constructs at the behavioral level; however, dual systems and related risk-taking theories are multi-levelled and propose that a developmental imbalance may be observed at a neural, cognitive, and behavioral level (Shulman et al. 2016). Future research will be helpful to establish whether similar subgroups to those in the present study are evident when using imaging and task-based measures of self-regulation and sensation-seeking.

It is important to note the limitations of this study. First, data came from a large longitudinal cohort study and thus only brief measures of sensation-seeking and self-regulation were available. These measures were adapted from a multi-dimensional measure of self-control that includes components that have been noted to map to sensation-seeking and self-regulation; however, further psychometric evaluation studies would be helpful to confirm that these successfully capture these constructs. At the same time, further conceptual work is required to provide clearer and more specific definitions of these constructs as they are used in the context of duals systems theories to optimize their measurement via questionnaires, tasks, and imaging measures. Future studies should aim to replicate the current findings using more comprehensive validated measures of sensation-seeking and self-regulation constructs. Second, delinquency was based only on self-reports, therefore, future research drawing on other data sources such as official records data will be beneficial to evaluate the extent to which results replicate when using alternative methods of capturing delinquency. Finally, latent class/mixture modeling techniques involve a number of decision-points that can affect the ultimate interpretation of findings and it is important to acknowledge the inherent subjectivity of the technique.

## Conclusion

Dual systems theory has been proposed as an explanation for an adolescent peak in offending; however, no study has yet examined whether a delinquency escalation developmentally tracks an imbalance between sensation-seeking and self-regulation. Using a longitudinal latent class growth analysis method, the current study’s results suggest that dual systems theory can explain a peak in offending behavior in adolescence; however, only for a small sub-group of youth accounting for just over 7% of the present normative sample (or 10% of the male sample). Most youth show no developmental imbalance in self-regulation and sensation-seeking traits nor an elevation of delinquency. This suggests that dual systems theory is better considered a developmental psychopathology model for a high-risk male group than a theory of normative development. Embedding dual systems theory within broader models that acknowledge the heterogeneity of sensation-seeking and self-regulation trajectories can provide a more accurate description of maladaptive risk-taking behaviors across development.
